# Early intervention, treatment and rehabilitation of employees with common mental disorders by using psychotherapeutic consultation at work: study protocol of a randomised controlled multicentre trial (friaa project)

**DOI:** 10.1186/s12889-021-11195-9

**Published:** 2021-06-22

**Authors:** Jeannette Weber, Peter Angerer, Lorena Brenner, Jolanda Brezinski, Sophia Chrysanthou, Yesim Erim, Manuel Feißt, Marieke Hansmann, Sinja Hondong, Franziska Maria Kessemeier, Reinhold Kilian, Christina Klose, Volker Köllner, Fiona Kohl, Regina Krisam, Christoph Kröger, Anja Sander, Ute Beate Schröder, Ralf Stegmann, Uta Wegewitz, Harald Gündel, Eva Rothermund, Kristin Herrmann

**Affiliations:** 1 Institute of Occupational, Social and Environmental Medicine, Centre for Health and Society, Medical Faculty, Heinrich-Heine-University Düsseldorf, Moorenstraße 5, 40225 Düsseldorf, Germany; 2 Research Group Psychosomatic Rehabilitation, Department of Psychosomatic Medicine, Center for Internal Medicine and Dermatology, Charité - Universitätsmedizin Berlin, corporate member of Freie Universität Berlin and Humboldt-Universität zu Berlin, Hindenburgdamm 30, 12203 Berlin, Germany; 3 Institute of Medical Biometry and Informatics, University Hospital of Heidelberg, Im Neuenheimer Feld 130.3, 69120 Heidelberg, Germany; 4 Department of Psychosomatic Medicine and Psychotherapy, University Hospital of Erlangen, Friedrich-Alexander University Erlangen-Nürnberg (FAU), Schwabachanlage 6, 91054 Erlangen, Germany; 5 Institute of Psychology, University of Hildesheim Foundation, Universitätsplatz 1, 31141 Hildesheim, Germany; 6 Institute of Medical Biometry and Statistics, Section of Health Care Research and Rehabilitation Research, Faculty of Medicine and Medical Center, University of Freiburg, Hugstetter Straße 49, 79106 Freiburg, Germany; 7 Department Psychiatry II, Section of Health Economics and Psychiatric Services Research, Ulm University, Lindenallee 2, 89312 Günzburg, Germany; 8 Federal Institute for Occupational Safety and Health (BAuA) Division 3 Work and Health Unit 3.5 Evidence-based Occupational Health, Workplace Health Management, Nöldnerstr, 40-42 10317 Berlin, Germany; 9 Department of Psychosomatic Medicine and Psychotherapy, Ulm University Medical Center, Albert-Einstein-Allee 23, 89081 Ulm, Germany

**Keywords:** Mental health, Workplace, Return to work, Psychotherapy, Depression, Self-efficacy, Sickness absence

## Abstract

**Background:**

Common mental disorders are one of the leading causes for sickness absence and early retirement due to reduced health. Furthermore, a treatment gap for common mental disorders has been described worldwide. Within this study, psychotherapeutic consultation at work defined as a tailored, module-based and work-related psychotherapeutic intervention will be applied to improve mental health care.

**Methods:**

This study comprises a randomised controlled multicentre trial with 1:1 allocation to an intervention and control group. In total, 520 employees with common mental disorders shall be recruited from companies being located around five study centres in Germany. Besides care as usual, the intervention group will receive up to 17 sessions of psychotherapy. The first session will include basics diagnostics and medical indication of treatment and the second session will include work-related diagnostics. Then, participants of the intervention group may receive work-related psychotherapeutic consultation for up to ten sessions. Further psychotherapeutic consultation during return to work for up to five sessions will be offered where appropriate. The control group will receive care as usual and the first intervention session of basic diagnostics and medical indication of treatment. After enrolment to the study, participants will be followed up after nine (first follow-up) and fifteen (second follow-up) months. Self-reported days of sickness absence within the last 6 months at the second follow-up will be used as the primary outcome and self-efficacy at the second follow-up as the secondary outcome. Furthermore, a cost-benefit assessment related to costs of common mental disorders for social insurances and companies will be performed.

**Discussion:**

Psychotherapeutic consultation at work represents a low threshold care model aiming to overcome treatment gaps for employees with common mental disorders. If successfully implemented and evaluated, it might serve as a role model to the care of employees with common mental disorders and might be adopted in standard care in cooperation with sickness and pension insurances in Germany.

**Trial registration:**

The friaa project was registered at the German Clinical Trial Register (DRKS) at 01.03.2021 (DRKS00023049): https://www.drks.de/drks_web/navigate.do?navigationId=trial.HTML&TRIAL_ID=DRKS00023049.

**Supplementary Information:**

The online version contains supplementary material available at 10.1186/s12889-021-11195-9.

## Background

Common mental disorders (CMDs) are one of the major public health problems worldwide with an estimated twelve-month global prevalence rate of 17.6% [1]. According to their high burden of disease [2], CMDs are one of the leading causes for sickness absence [3] and go along with high risks of early retirement [4]. Prevention, early treatment and well-concerted reintegration after sickness absence might act as key factors to reduce the risk of chronicity of CMDs, long and recurrent sickness absence and early retirement due to CMDs. However, even in high-income countries, large treatment gaps were reported. For example, only 5 % of individuals with anxiety disorders and 22% of individuals with major depressive disorders are estimated to receive adequate treatment [5, 6]. On the one hand, reasons for this treatment gap are supposed to be person-related including lack of perceived need for treatment [5] and fear of stigmatisation [7, 8]. On the other hand, reasons are related to the health-care system itself including long waiting times on therapy [9–11].

Psychotherapeutic consultation at work is a new and low threshold concept for early prevention and treatment of employees with symptoms of CMDs [12, 13]. It might offer first consultation, diagnostic identification, referral to care as usual (CAU), proceeding psychotherapy, and therefore possibilities to tackle treatment gaps regarding mental health [14]. First evidence suggests that psychotherapeutic consultation at work significantly reduces depressive symptoms and anxiety and improves work ability [15]. In addition, extrapolation suggests that screening of CMDs and subsequent psychotherapeutic treatment at work is cost effective [16]. Furthermore, psychotherapeutic consultation at work can serve as a model for close collaboration between occupational health physicians, psychotherapists and other mental health care providers [17]. Such collaboration has been regarded as an additional key factor to improve treatment and return to work (RTW) of employees with CMDs [18]. For example, within a previous randomised controlled trial (RCT), referral of employees with depression to psychiatric care by occupational health physicians was combined with collaboration between those health care providers during vocational reintegration. This intervention was found to be associated with faster RTW compared to CAU [19]. More successful and sustainable RTW might further be achieved by combining CAU and work-related psychotherapy as evidenced by previous research [20–26]. Concepts of work-related psychotherapy were most often developed to support vocational reintegration of employees being sick-listed due to CMDs, including - inter alia - work-related assessments, assistance in drawing RTW plans and contacting employers and occupational physicians as well as evaluation of RTW steps [19–24]. However, work-related psychotherapy could also contribute to reduction of depressive symptoms and improvement of work ability at earlier stages [27, 28].

Particularly a combination of a) prevention, b) early treatment, c) work-related psychotherapy and d) collaboration between key (mental) health care professionals might help to tackle the reported treatment gap and therefore chronicity, long and recurrent sickness absence and early retirement due to CMDs. However, within occupational settings, previous studies have mainly investigated the effectiveness of interventions addressing either one or two of those aspects (e.g. [15, 21, 22, 24, 27, 28]). Furthermore, study samples were often small including only one or two companies and cost-benefit assessments are seldom performed (e.g. [15, 19, 25, 27, 28]). RCTs testing the effectiveness of more comprehensive care models in small, middle as well as large-sized companies are scarce.

This two-arm randomised controlled multicentre trial - called early intervention in the workplace (German: Frühe Intervention am Arbeitsplatz, friaa) - therefore aims to evaluate psychotherapeutic consultation at work, which in this study consists of a tailored, module-based and work-related psychotherapeutic intervention. The intervention will thereby combine the four aspects of prevention, early treatment, work-related psychotherapy and collaboration between key (mental) health care professionals. Employees with symptoms of CMDs will be randomised with 1:1 allocation to an intervention group receiving psychotherapeutic consultation at work or to a control group receiving CAU. Participants’ outcome data of both groups will be collected nine (first follow-up) and fifteen (second follow-up) months after enrolment.

The primary aim is to investigate whether the intervention is superior to CAU regarding reduction of sickness absence within the last 6 months at the second follow-up among employees with symptoms of CMDs.

The secondary aims are to investigate whether the intervention is superior to CAU regarding

- levels of occupational self-efficacy at the second follow-up among employees with symptoms of CMDs
- a health economic evaluation related to costs of CMDs for social insurances and companies at second follow-up

## Methods/design

### Participants, interventions, and study variables

#### Study setting and recruitment

Participants will be recruited from small, middle and large-sized companies being located around five study centres in Germany (in and around Ulm, Düsseldorf, Teltow, Hildesheim and Erlangen) over a period of twelve months, starting in September 2021. Psychotherapeutic consultation at work will be established in close collaboration with occupational health services in or outside of business premises based on preferences of participating companies. Employees with psychological complaints will then be transferred to psychotherapeutic consultation at work by occupational health physicians, supervisors or by self-assignment. Each employee who is transferred to psychotherapeutic consultation at work will receive diagnostic assessment regarding mental health and recommendations for further procedures in CAU (Module 1-A). They will be screened for eligibility criteria and if eligible, will be asked to participate by written informed consent. Participants will then be randomised to the intervention group or control group with 1:1 allocation. Participants not meeting the eligibility criteria will get the according recommendation to CAU. The intervention modules will be accompanied by setting up a network of occupational health services, psychotherapists conducting the psychotherapeutic consultation, clinics for psychosomatic medicine and rehabilitation centres depending on local conditions. Communication between those (mental) health care providers will be established in the form of standardised handovers. Furthermore, educational programs will be offered to all occupational physicians and psychotherapists taking part in the intervention.

#### Eligibility criteria

Inclusion criteria:

- written informed consent to participate in the study
- aged 18 years or above
- sufficient knowledge of the German language to participate in the study
- employment for at least 15 h per week in participating companies
- diagnosis of a CMD according to the International Statistical Classification of Diseases and Related Health Problems (ICD-10 [29]) including unipolar depressive disorders (F32-F34), anxiety disorders (F40, F41), stress-related and somatoform disorders (F42, F43, F45, F48.0) and non-organic sleep disorders (F51) or subclinical symptoms of psychosomatic disorders without ICD-10 diagnosis
- symptoms of psychosomatic disorders without ICD-10 diagnosis measured by the Global Assessment of Functioning Scale (GAF Scale < 81, [30, 31]).

Exclusion criteria:

- unique or main diagnosis of substance abuse (F10–19), schizophrenia, psychosis (F20-F29) or organic psychiatric disorders (F00-F09)
- severe and unstable somatic health condition (e.g. cancer)
- current psychotherapeutic treatment
- application for retirement pension

#### Intervention

All sessions of psychotherapeutic consultation at work will be conducted by licenced psychological or medical psychotherapists or by psychological or medical psychotherapists being in an advanced state of postgraduate training for psychotherapy. The intervention consists of three modules. The first module includes one session of basic diagnostic assessment on mental health (Module 1-A) and one session of work-related diagnostic assessment (Module 1-B). Whereas Module 1-A will be offered to the intervention and control group, Module 1-B and all other modules will exclusively be offered to the intervention group. Based on mental health assessment, participants of the control group will receive recommendations for treatment in CAU (e.g. in- or outpatient acute care, inpatient vocational rehabilitation). Participants of the intervention group will be transferred to CAU if outpatient work-related treatment would not sufficiently cover the individual need for treatment or will receive Module 2. In case participants of the intervention group are transferred to CAU, potential waiting times for treatment will be bridged by Module 2. Module 2 will include up to ten sessions of work-related psychotherapeutic consultation or treatment. Participants of the intervention group with an inability to work will receive further psychotherapeutic consultation during their RTW process in up to five sessions during Module 3. Participants of the intervention group may thus receive up to 17 sessions of psychotherapeutic consultation (Module 1-A: one session; Module 1-B: one session; Module 2: ten sessions; Module 3: five sessions). For both, the control and intervention group, a medical report will be prepared. Participants of the intervention group who received up to four sessions of psychotherapeutic consultation and participants of the control group will receive a follow-up call by study therapists twelve weeks after their last visit. In this call, participants will be asked on how they have coped so far and whether further help regarding treatment in CAU is needed. For all other participants, an equal follow-up will be performed during their last session of psychotherapeutic consultation.

**Fig. 1:**
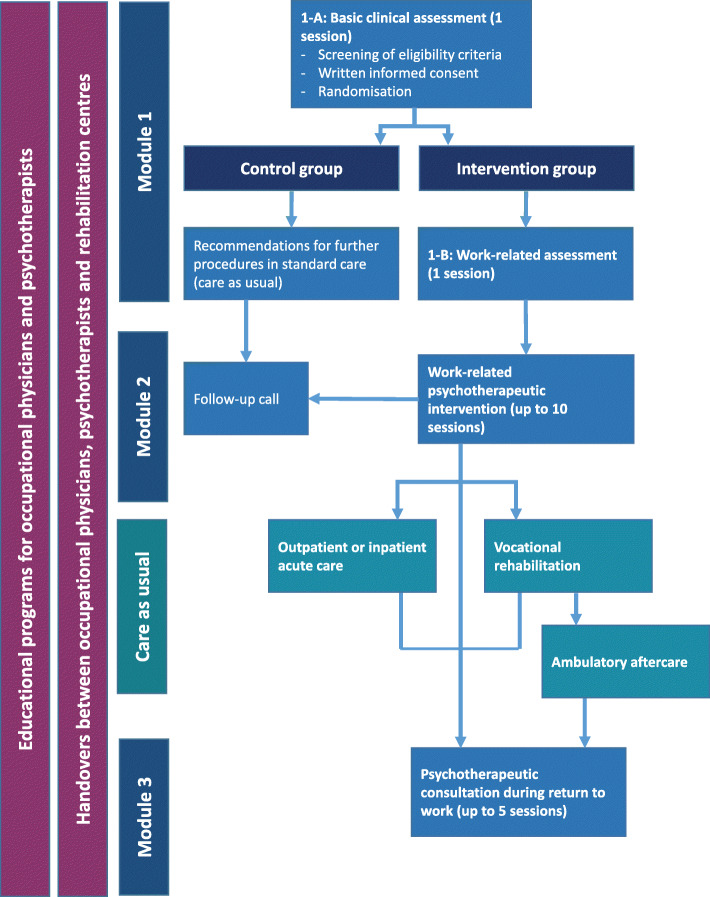
Overview on study intervention

A schematic overview of the intervention is given in Fig. 1 and all its components are further described below.

##### Module 1-a. basic clinical assessment

- Medical and psychosomatic history
- International Neuropsychiatric Interviews (MINI [32])
- Screening of eligibility and inclusion in the study
- Randomisation of participants into intervention or control group
- Recommendations for further procedures in CAU (e.g. in- or outpatient acute care, inpatient vocational rehabilitation)
- Participants of the intervention group may further be referred to the second module of the intervention.

##### Module 1-B. work-related assessment

- Work-related assessment including work history, job description, work tasks, work relationships and person-job fit as described elsewhere [25, 33]. If possible, information on those aspects being provided by occupational physicians will also be taken into account
- Analysis of working conditions, social conflicts and social-therapeutic aspects including general social problems or concealed resources as described by Köllner [34]

##### Module 2. Work-related psychotherapeutic intervention

During module 2, an adaption of the manual from Bode et al. [33] will be used to conduct work-related psychotherapeutic consultation. This will include

- Psychoeducation providing information on the relations between work and mental health
- Formation of an individual exploratory model of current psychosomatic symptoms. In this model, stressors and resources at work will be included
- Using communication techniques to increase, maintain or restore motivation to work
- Defining achievable tasks as well as focusing on achievements and resources at work to promote self-efficacy
- Discussing problems at work, elaboration of solution approaches and searching for contact persons at work to implement those solution approaches
- Evaluation and treatment of work-related symptoms (e.g. anxiety or overload) by using cognitive restructuring methods
- Implementation of work-related cognitive-behavioural therapeutic treatment measures: resource activating measures, relaxation techniques that can be used at the workplace, coaching of social competences, communication exercises, acceptance of unchangeable situations, clarification of intrapersonal conflicts, promotion of acceptance and emotional competence
- Detection of work-related risk factors for development and deterioration of CMDs (e.g. shift work) and giving recommendations of setting-based prevention measures to minimise them

Depending on severity of symptoms, psychotherapeutic consultation during module 2 may either include more curative or preventive aspects. Therefore, module 2 is subdivided into module 2-A comprising work-related short-term psychotherapy and module 2-B comprising work-related prevention. Module 2-A contains both disorder specific psychotherapeutic interventions and the above mentioned work-related interventions. Participants with an ICD-10 diagnosis will thus receive module 2-A and participants with subclinical psychosomatic symptoms without an ICD-10 diagnosis will receive module 2-B.

##### Module 3. Psychotherapeutic consultation during RTW

RTW should be prepared and implemented in consideration of the four phase model of vocational reintegration [35]. Therapeutic sessions of module 3 will be offered to participants with prior sickness absence to support RTW. This will include the implementation of therapeutic issues and strategies from module 1-B and 2. Furthermore, module 3 will comprise

- Psychotherapeutic support and evaluation of each reintegration step (e.g. discussion of successful and unsuccessful aspects or needs for adjustments of the reintegration plan) as described by Bode et al. [33]
- If the patient provides informed consent, study psychotherapists will be involved within the occupational process of reintegration and will collaborate with responsible RTW actors (e.g. occupational physician)

Weekly sessions will be intended.

##### Handovers

- Accompanying all modules of the intervention
- Standardised forms for communication between occupational physicians, psychotherapists and other care providers
- Handovers will only be performed with patient’s permission under consideration of medical confidentiality and data protection legislation

##### Contents of educational programs for psychotherapists and occupational physicians

- Applications for rehabilitation measures in the German health care system
- Draft of medical reports to increase efficient communication between health care providers
- Information on aims, processes and regulations of prevention and rehabilitation offers of sickness and pension insurances in Germany
- Culture-specific approaches during treatment according to Erim et al. [36]
- Additional contents within the educational program for psychotherapists: training in the intervention modules and basic knowledge about occupational health care
- Additional contents within the educational program for occupational health physicians: Psychotherapeutic primary care

#### Discontinuation and modifications of the intervention protocol

The intervention might be discontinued if symptoms decrease and there is no indication for further treatment. If an appointment has been missed, efforts will be made to contact the participant to schedule a new appointment. However, in case that a participant refuses further treatment, the intervention will be discontinued and participants will be asked for their reasons for refusal. If symptoms increase, the psychotherapist and possibly supervisor (i.e. a versed psychotherapist) will weight up further treatment needs. They might schedule a higher frequency of therapy sessions - but no more than 10 sessions of module 2 and 5 sessions of module 3 - or might refer participants to inpatient care. In each case, participants will remain in the study and will be reminded of the follow-up measurements in their last therapy session.

If participants withdraw their informed consent to participate in the study, further therapy sessions will be cancelled. In this case, follow-up measurements will not be performed. If participants also withdraw their informed consent of data storage, collected data of those participants will be deleted and not be included within data analysis.

In general, concomitant care and interventions (e.g. outpatient psychiatric or neurological treatment, somatic treatment, non-medical practitioner treatment, psychological coaching, use of helplines and helpdesks at work) are not prohibited during the trial. Moreover, module 2 aims to bridge waiting times on CAU and therefore also outpatient psychotherapeutic and inpatient psychotherapeutic, psychiatric or rehabilitation treatment are not prohibited during the trial. However, the intervention will be terminated or paused during CAU. Employees receiving psychotherapeutic treatment before starting the intervention are excluded from the study.

In general, discontinuation and all deviations of the intervention protocol will be documented.

#### Adherence to the study protocol

Several strategies will be implemented to ensure adherence to the intervention protocol. First, psychotherapists will be trained in the intervention modules during the two-day educational program. Second, psychotherapists will be constantly supervised by a versed therapist and a scientific assistant from the study. Third, therapists will be instructed to record defined treatment sessions on video. The manual from Bode et al. (2017) served as a basis for selecting important interventions [33]. For the evaluation of treatment integrity only those treatment sessions that contain the interventions will be recorded and analysed. Assignment to intervention modules, number of sessions in each module and session dates will be documented and completeness of data will be constantly monitored during the intervention by the psychotherapists and scientific assistants. Furthermore, participants will be regularly reminded of follow-up measurements.

#### Outcomes

##### Primary outcome

Days of sickness absence within the last 6 months at second follow-up will serve as the primary outcome. As in comparable previous studies [25, 37], days of sickness absence is chosen as the primary outcome due to its economic value and because it is a relevant parameter of psychotherapeutic and psychiatric rehabilitation measures. Days of sickness absence within the last 6 months will be collected as a self-reported item by standardised paper-and-pencil or online questionnaires during baseline and during follow-ups. This item was self-developed for this study. Participants will be asked whether they were absent from work for health reasons within the last 6 weeks with response options “yes” and “no”. If participants ticked “yes”, they will be asked about the number of days of sickness absence within the last 6 months excluding regular non-working days.

##### Main secondary outcomes

Self-efficacy at the second follow-up will serve as a secondary outcome. Self-efficacy is chosen as a secondary outcome due to its predictive value of successful RTW [38–40]. Information on self-efficacy will be collected by standardised paper-and-pencil or online questionnaires during baseline and during follow-ups.

To measure self-efficacy regarding RTW, a German version of the validated return-to-work self-efficacy scale (RTW-SE [41]) will be used. The RTW-SE scale consists of eleven items with a six-point rating scale from 1 = “totally disagree” to 6 = “totally agree”. Example items are “I will be able to cope with setbacks” and “I will be able to concentrate on my work”.

Occupational self-efficacy will be measured by the German version of the validated short form of the Occupational Self-Efficacy Scale [42]. The Occupational Self-Efficacy scale consists of six items with a six-point rating scale from 1 = “not at all true” to 6 = “completely true”. Example items are “I can remain calm when facing difficulties in my job because I can rely on my abilities” and “Whatever comes my way in my job, I can usually handle it”.

Both scales were shown to be reliable by previous research with good to excellent internal consistencies [41, 42]. Therefore, mean scores will be calculated for both scales.

##### Health economic evaluation

Health economic evaluation will be conducted by means of an incremental cost-utility analysis (ICUA) following the net benefit approach [43, 44] from societal perspective [45]. Incremental cost utility ratios (ICUR) will be estimated for estimating the maximum willingness to pay (MWTP) needed for the gain of one quality adjusted life year (QALY) by providing the intervention in comparison to CAU [43, 44]. Comprehensive assessment of health service use and costs will be performed by means of the German version of the client sociodemographic and service receipt inventory (CSSRI [46, 47]) adjusted for CMDs. QALYs will be estimated by means of the EuroQol (EQ-5D-5L [48]) using the German value set [49, 50].

#### Additional data

Additional data will be collected either by standardised interviews conducted by the study psychotherapists or by standardised paper-and-pencil or online questionnaires. For most variables previously published scales will be used. An overview of additional study variables and relevant references is given in Table 1. Scales not being previously published (i.e. days of sickness absence and sociodemographic variables) are provided as supplemental material.

**Table 1:** Overview on additional study variables

Variable	Participants	Method
*Interview data*		
Global Assessment of Functioning	All	Global Assessment of Functioning Scale (GAF [30, 31])
Diagnosis	All	Derived from International Neuropsychiatric Interviews (MINI [32])
Indication of treatment	All	
Childhood trauma	All	Childhood trauma screener [51]
Work-related diagnostics	Intervention group	E.g. work history, job description, work tasks, work relationships, person-job fit [25, 33]
*Questionnaire data*		
Sociodemographic variables I	All	Age, gender, work experience
Sociodemographic variables II	All	Marital status, profession, weekly working hours, shift work, leadership position, company size, professional perspective, physical illness
Migration status	All	Basic set of indicators for mapping migrant status; German nationality, parental German nationality [52]
Demands of immigration	Participants with migrant background	Demands of Immigration Scale (DIS [53]
Mental health - depression	All	Depression items of the Patient-Health Questionnaire (PHQ-9 [54–56])
Mental health - anxiety	All	Generalized Anxiety Disorders (GAD-2 [57])
Mental health – somatoform disorders	All	Somatic Symptom Scale 8 (SSS-8 [58])
General health status	All	General health status item of the Veterans RAND 12 (VR-12 [59])
Cultural adaptation	Participants with migrant background	Frankfurt acculturation scale (FRAKK [60, 61])
Work productivity and activity impairment	All	Work productivity and activity impairment Questionnaire (WPAI [62])
Subjective work ability	All	First item of the Work ability index (WAI [63, 64])
Work role functioning	All	Work Role Functioning Questionnaire (WRPQ 2.0 [65]) – short version
Psychosocial working conditions	All	Third version of the Copenhagen Psychosocial Questionnaire (COPSOQ [66, 67]) including scales of job demands, control, development opportunities and social support
Psychosocial safety climate	All	Short version of the Psychosocial Safety Climate Questionnaire [68]
Personality Functioning	All	German version of the Level of Personality Functioning-screener brief form (LPFS-BF 2.0 [69, 70])
Integrated care	All	Modified version of a questionnaire on experiences on integrated care including scales of general coordination and coordination within and between care teams [71]

#### Participant timeline

The schedule of the study including enrolment, intervention and data assessment is given in Table 2.

**Table 2:**
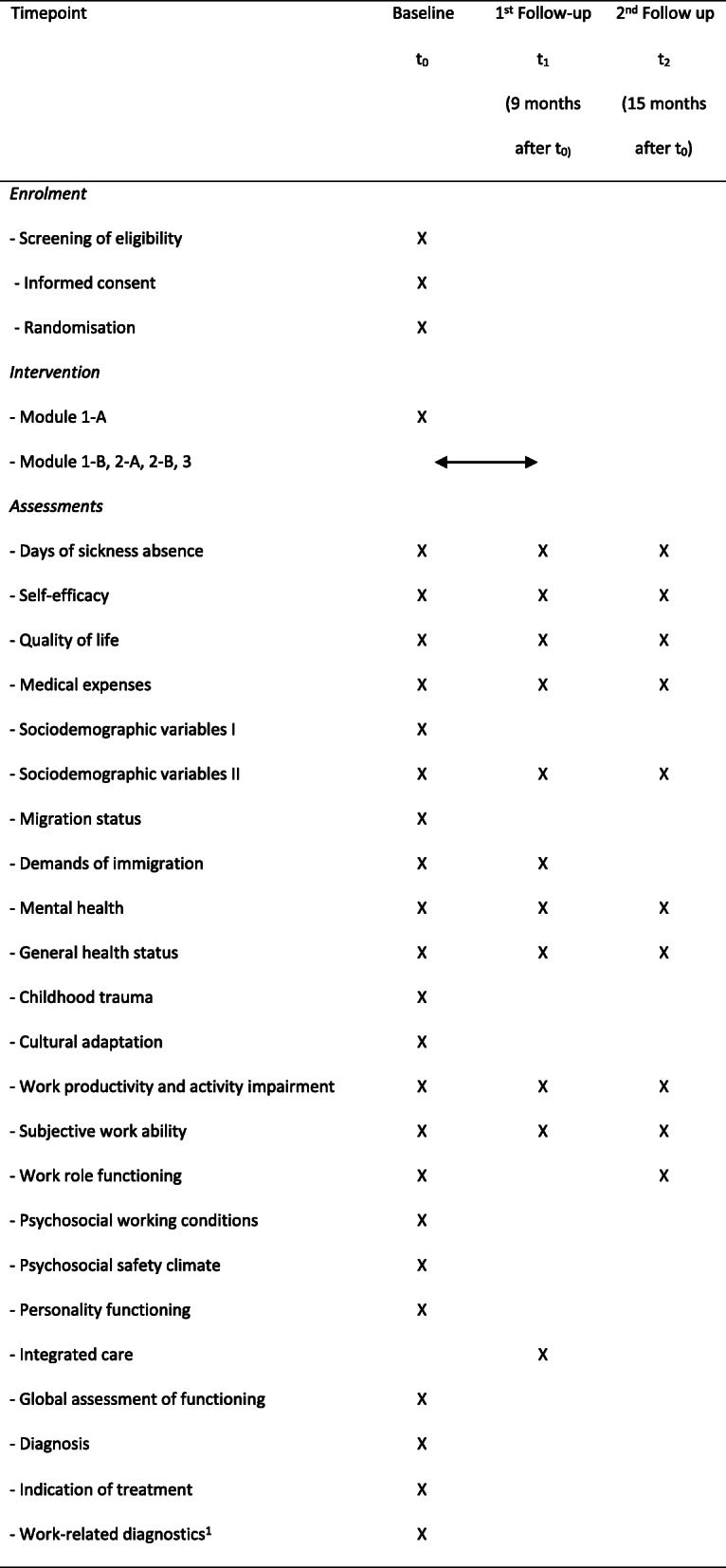
Schedule of the study

^1^ Work-related diagnostics will be performed during module 1-B

### Diversity issues

In order to meet the ethnic diversity of the insured, the migration background will be documented and analysed as a sociodemographic variable. In addition, the degree of cultural adjustment and post-migratory stress will be assessed with specialized questionnaires [53, 60, 61]. Since studies have shown that migrants benefit less from psychotherapy than natives [72], training (and supervision if needed) regarding culturally sensitive methods will be provided to optimize outcomes for subjects with a migrant background.

### Assignment of interventions

#### Allocation

Eligible participants will be allocated concealed by randomisation using a centralized web based tool (www.randomizer.at) after Module 1-A. Stratification by centre, days of sickness absence within the last 6 months (< 21 days vs. ≥ 21 days) and level of depression measured by the Patient Health Questionnaire-9 (PHQ-9 [54, 55]; ≤ 10 vs. > 10) using block randomisation of variable sizes will be performed.

#### Blinding

Participants will be blinded regarding study hypotheses but it will not be possible to blind them or study psychotherapists to allocation for obvious reasons. Furthermore, all variables at baseline will be collected prior to allocation to minimise reporting and selection bias.

### Data management and analysis

#### Data management

An electronic case report form (eCRF) will be used for data collection. Data from self-reported questionnaires will be collected web-based electronically or paper-based. Paper-based data will be entered into the eCRF by a study assistant. Paper-and-pencil questionnaires will be stored in locked filing cabinets and electronic data will be stored on secured servers. To assure a safe and secure environment for data acquired, data transmission is encrypted with secure socket layer (SSL) technology. Only authorized users are able to enter or edit data and access is restricted to data of the patients in the respective centre. All changes to data are logged with a computerized timestamp in an audit trail. To guarantee high data quality, data validation rules will be defined in a data validation plan. Completeness, validity and plausibility of data will be checked in time of data entry (edit-checks) and using validating programs, which will generate queries. The investigator or the designated representatives are obliged to clarify or explain the queries.

If no further corrections are to be made in the database, eCRF data will be locked. Data will finally be downloaded and used for statistical analysis. All data management procedures will be conducted according to written defined standard operating procedures that guarantee an efficient conduct complying with good clinical practice.

Except contact details of participants, all data will be pseudonymised. Contact details will be stored locally in the study centre in a separate document and are only accessible for authorised staff of the local study centre.

#### Sample size calculations

Sample size calculations were performed with PASS Version 16.0.3. Congruent with results of a recent uncontrolled trial, we expect that participation in psychotherapeutic consultation at work may reduce days of sickness absence from 36 days/6 months to 26 days/6 months at follow-up with a standard deviation of 35 days/6 months for both time points [73]. For the control group, we expect that days of sickness absence will increase from 36 days/6 months to 42 days/6 months due to the lack of work-related treatment in CAU [74] and possibly deterioration of health. The same standard deviation of 35 days/6 months will be expected for the control group. A negative binominal distribution of days of sickness absence will be expected in accordance with previous research [73]. Following those expectations, a dispersion parameter of 2.12 was calculated. To detect this difference with a power of 80% and assuming a two-tailed level of significance of 5%, a sample size of 310 participants with 155 participants for each group is required. Assuming dropout rates of 40% [15], 520 participants shall be recruited for the study in equal proportions per study centre.

#### Statistical methods

The treatment effect on the primary outcome (days of sickness absence within the last 6 months at the second follow-up) will be analysed using a mixed negative binomial regression model. Treatment group, days of sickness absence within the last 6 months before baseline, gender, age, subjective work ability at baseline as well as the centre will be included as covariates and the respective company as random effect. The primary analysis will be conducted based on the full analysis set according to the intention-to-treat (ITT) principle (i.e. all patients will be analysed in the group they were randomised to).

Missing data for the primary and secondary outcome analysis is assumed to be “missing at random” and replaced using multiple imputation based on predictive mean matching using the covariates of the primary and secondary outcome analysis as potential predictors [75].

As a sensitivity analysis, an evaluation based on the per-protocol (PP) population will be performed. The PP set consists of all participants without major protocol violations. For this analysis, no imputation of missing data will be performed.

In case of significance of the ITT analysis of the primary outcome (two-sided confidence level of 5%), a hierarchical linear mixed model will be used to analyse treatment effects on self-efficacy. This model will include treatment group, self-efficacy at baseline, gender, age, subjective work ability at baseline as well as the centre as covariates and company as random effect. Applying this hierarchical testing strategy, the overall type I error rate will still be controlled.

If no significance of the ITT analysis is achieved, the results of this analysis will be interpreted only in a descriptive sense.

Furthermore, subgroup analyses will be performed to evaluate the role of additional variables (e.g. indication of treatment, type of treatment, symptom severity) on treatment effects on the primary and secondary outcome.

Further collected data (see Table 1) will be analysed using appropriate descriptive methods.

Details of the statistical analysis will be further determined in a statistical analysis plan which will be written before database closure.

All those analyses will be conducted using SAS 9.4 or higher.

For the health economic evaluation, stochastic uncertainty of ICUR will be estimated by means of non-parametric bootstrapping with 2000 replications [76]. ICUR will be interpreted based on the cost-effectiveness plan [43]. MWTP necessary for the gain of one QALY by the implementation of the intervention in comparison to CAU will be estimated for the threshold range between 0 and 125.000€ [77] based on the cost effectiveness acceptability curve [43, 44, 76]. The health economic evaluation will be conducted using STATA 16.

### Monitoring

#### Data monitoring

An independent data monitoring committee (DMC) will be established composed of two clinical professionals and one biometric professional. The DMC will be regularly informed about the course of the trial and all safety issues. Furthermore, the DMC will be asked for advice whether to continue, modify or stop the trial.

#### Harms

Occurrence of adverse events will be documented. Although adverse events are rare during psychotherapy [78], they cannot be completely ruled out. Adverse events may include lack of treatment results, occurrence of new symptoms, increasing symptoms, strains in therapist-patient relationship, strains or changes in work, family or other social relationships, stigmatisation and development of pathological dependency to the therapist [79–81]. In case of adverse events, the psychotherapist and possibly supervisors will weight up further treatment needs as described under *Discontinuation and Modifications of the intervention protocol*. Furthermore, measures will be taken to avoid adverse events including i) the educational programme for psychotherapists to increase awareness of adverse events, ii) supervision of psychotherapists and iii) progress monitoring as described by Rozental [82].

### Formative evaluation

A formative evaluation of the intervention using qualitative methods will be realised. Data will be collected in focus groups and individual interviews. The focus groups and interviews will be recorded and transcribed.

First, prior to the intervention, two focus groups (approximately eight participants per group) are conducted with a) health care professionals and b) employees who have experience with psychotherapeutic consultation and return to work. These focus groups will be analysed using qualitative content analysis with regard to expectations of the participants, facilitating and hindering factors related to early prevention in company settings and future implementation of the intervention.

Second, four narrative focus groups (approximately eight participants per group) will be conducted with a) organisational and b) external experts in the RTW process before and after the implementation of the intervention. The focus groups will be analysed using qualitative content analysis. Expectations of the intervention, facilitating and hindering factors as well as valuation of the intervention will be examined. In addition, knowledge and handling of mental illness will be reconstructed.

Third, a subsample of 20 participants of the intervention group will be questioned in individual narrative interviews at two time points: at the end of the intervention and approximately 6 months later after (gradual) RTW. In more detail, the following subsamples of participants will be interviewed:

- Up to six participants who only received module 2-A and/or 3
- Up to five participants who received module 2-A as well as outpatient or in patient acute treatment and module 3
- Up to five participants who received module 2-A, vocational rehabilitation as well as module 3
- Up to four participants who only received module 2-B.

In addition, five participants of the control group will be narratively interviewed 6 months after returning to work. All individual interviews will be analysed using the Documentary Method of Interpretation [83]. This method not only allows to identify reflexive knowledge, but above all to reconstruct tacit knowledge, knowledge of action and experiences. Participants’ expectations of the intervention, experiences and behaviour in the process of intervention, as well as the effect of the intervention on RTW will be reconstructed. In addition, interactions between intervention modules and contextual conditions as well as facilitating and hindering factors will be analysed. Furthermore, effects of the intervention on self-management and self-efficacy as well as the valuation of the intervention by the participants will be examined.

Fourth, based on an overarching case comparison, similarities and differences will be identified in focus groups and individual interviews (referring to the interest in knowledge as mentioned above). The comparison allows evaluation of the intervention beyond the individual case. In summary, the formative evaluation aims to identify facilitating and hindering factors concerning the various intervention modules and in relation to different contextual conditions such as company size and structure.

## Discussion

Worldwide, a treatment gap for CMDs has been described [5, 6]. Inadequate and late treatment may increase the risk of chronicity of CMDs [84] and therefore might contribute to long and recurrent sickness absence and premature retirement [3, 4]. The workplace and its working conditions may contribute beneficially or adversely to the development of CMDs [85–87]. At the same time, the workplace might function as a setting to enable utilization of mental health care interventions by reaching a high number of individuals suffering from CMDs [12].

This study protocol therefore describes a multicentre RCT, which aims to test psychotherapeutic consultation at work. In this study, psychotherapeutic consultation at work consists of a tailored, module-based and work-related psychotherapy. It especially aims to reach patients at early disease stages as well as patients with subclinical symptoms [17]. Due to long waiting times for psychotherapeutic offers in the German health care system [10], we expect that patients of the control group are treated at later time points. We therefore expect that they also report more days of sickness absence than patients of the intervention group. Besides disorder-specific psychotherapy, the intervention group will also receive work-related psychotherapy and elaborate cooperation between mental health care actors. Consistent with previous research [19, 20, 22, 37, 88], those aspects may result in additional reduction of sickness absence in the intervention group. In case that the intervention is shown to be effective to reduce sickness absence and referring to the high prevalence and burden of disease of CMDs [1, 2], implementation of psychotherapeutic consultation at work into practice could have large public health relevance.

If the intervention is shown to be effective to reduce days of sickness absence, we expect that direct and indirect costs related to CMDs are reduced. An additional health economic evaluation will therefore be performed to test whether the intervention is actually cost effective. Previous health economic evaluations of similar interventions [16, 20] may raise expectations of a positive evaluation. In this case, implementation of psychotherapeutic consultation at work might be of economic relevance by relieving financial pressure on health and pension insurances and reducing costs related to loss of productivity due to CMDs.

In Germany, large-scale implementation of psychosomatic consultation at work might be realized by joint financing by health and pension insurances. In accordance with German Social Law, module 1 and 2 might be financed by health insurances as special forms of health care (§140a SGB V) and module 3 might be financed by the German pension insurance as employment participation benefits (§49 SGB IX). Besides publication of research results in international research journals, a manual of the intervention will be developed and made available to the public to support implementation of psychotherapeutic consultation at work into practice.

### Strengths and limitations

Strengths of this study will include the randomised controlled study design, the large sample size and recruitment of employees from various companies. By including small, middle and large-sized companies in public and private industry around five study centres in Germany, we aim to improve external validity of our study results. However, external validity outside Germany will be limited due to differences in health care systems.

The fact that the control group will receive a first session of psychotherapeutic consultation at work including basic clinical assessment and recommendations for CAU within the German health care system might be perceived as a second limitation of this study. With this approach, treatment of the control group does not completely resemble CAU. Psychosomatic consultation at work has been shown to reach patients at earlier disease stages than CAU [12]. The control group could consequently receive earlier diagnoses and due to recommendations could also receive earlier treatment in CAU compared to usual study-independent conditions. This may further lead to faster recovery, better prognosis, prevention of chronicity [84, 89] and subsequently to reduced days of sickness absence. Intervention effects could therefore be underestimated. However, this approach is chosen for practical and ethical reasons. First, eligibility to participate in the study will be evaluated during basic diagnostics. Second, acceptance of the study by companies and their employees is thought to be increased by the fact that each study participant will receive at least one session of psychotherapeutic consultation at work. Third, giving recommendations for further treatment after diagnosis of health issues should be self-evident for ethical reasons.

Recruitment of study participants might be complicated by stigmatisation of mental disorders [7, 8], which is especially present in workplace settings [90–92]. Employees might fear that their colleagues, supervisors and employers could find out about visits to psychotherapeutic consultation at work and might therefore reject study participation [93]. To prevent fear of being seen by colleagues and supervisors when visiting psychotherapeutic consultation at work, efforts will be made to provide consultation in secure and confidential facilities. Furthermore, confidentiality of study participation towards third parties will always be guaranteed.

## Conclusion

This protocol describes a multicentre RCT to test intervention effects of a tailored and module-based type of psychotherapeutic consultation at work. By providing prevention, early treatment, work-related psychotherapy and inducing collaboration between key (mental) health care professionals, the interventions aims to reduce days of sickness absence and increase self-efficacy of employees with CMDs. Referring to the high prevalence of CMDs [1], their large burden of disease [2] and economic impact on labour markets and social security systems [94], a positive evaluation of the intervention and subsequent implementation into practice could be of large public health and economic relevance.

## Supplementary Information

**Additional file 1.** Study questionnaire for sociodemographic variables and days of sickness absence.

**Additional file 2.** SPIRIT 2013 checklist.

## Data Availability

Not applicable.

## Abbreviations

CAU: Care as usual
CMD: Common mental disorder
CSSRI: Client Sociodemographic and Service Receipt Inventory
DMC: Data monitoring committee
ICD: International Statistical Classification of Diseases and Related Health Problems
ICUA: Incremental cost-utility analysis
ITT: Intention-to-treat
MWTP: Maximum willingness to pay
PP: Per-protocol
RCT: Randomised controlled trial
RTW: Return to work
RTW-SE: Return-to-work self-efficacy scale
QALY: Quality adjusted life year
