# Supplementary material for: Early intervention, treatment and rehabilitation of employees with common mental disorders by using psychotherapeutic consultation at work: study protocol of a randomised controlled multicentre trial (friaa project)
Source: BMC Public Health. 2021 Jun 22;21:1187. doi: 10.1186/s12889-021-11195-9 (PMC8218449; doi:10.1186/s12889-021-11195-9)
Supplement: Supplementary file 1 — Additional file 1. Study questionnaire for sociodemographic variables and days of sickness absence. [file 12889_2021_11195_MOESM1_ESM.pdf]

Additional file 1 Study questionnaire for sociodemographic variables and days of sickness absence

Personal details

1. Sex 1☐ male 2☐ female 3☐ divers

2. Age _____ **years**

**3. Marital status** (please tick only one box)	1 ☐ married/ in permanent relationship<br>2 ☐ single	3 ☐ divorced<br>4 ☐ widowed

Occupational details

**4. a) What best describes your current employment situation?** (please tick only one box)	1 ☐ Employed fulltime or parttime<br>2 ☐ trainee<br>3 ☐ voluntary work (without payment)<br>4 ☐ protected activity<br>5 ☐ unemployed/seeking employment since______
**4. b) If 4.a is not applicable: What best describes your social situation?** (please tick only one box)	6 ☐ Student<br>7 ☐ Homemaker<br>8 ☐ Pension (including early retirement)<br>9 ☐ occupational disability since_______<br>10 ☐ maternity or paternal leave or other leave of absence<br>11 ☐ other, namely<br>______________________________
**4. c) If employed, for how many hours are you working?**	fulltime, hours/week:____________<br>part-time, hours/week:____________
**4. d) For how many days per week are you working?**	days/weeks:_____________________
**5. Are you doing shiftwork?**	1 ☐ no<br>2 ☐ yes including night work

	3 ☐ yes without night work
**6. How long have you been working?**	Since |__|__| years or since the years |__|__|__|__|

7. Which professional group do you currently belong to (please tick only one box)**?**

1☐ Unskilled work
2☐ Skilled worker
3☐ Foremen
4☐ Self-employed
5☐ Executing employee (e.g. secretary)
6☐ Mid-level employee (e.g. clerk, assistant)
7☐ Senior/executive employee (e.g. department head, team leader)
8☐ Executing civil servant (e.g. secretary)
9☐ Mid-level civil servant (e.g. teacher)
10☐ Senior/executive civil servant (e.g. department head)
11☐ Other: ________________________________

8. Do you have a management position?

1☐ no
2☐ yes
If yes, for how many employees are you responsible for? Number: _______

9. How big is the company you are working for (please only tick one box. If unknown, please tick what is most likely to apply)?

1☐ Large-sized company (>1000 employees)
2☐ Large-sized company with less employees (about. 250-1000 employees)
3☐ Middle-sized company (about 50-249 employees)
4☐ Small-sized company (about 10-49 employees)
5☐ Microenterprise (about 2-9 employees)
6☐ One-man business

10. Have you been absent from work for health reasons within the last 6 months?

1 ☐ no
2 ☐ yes. If yes, number of days of sickness absence within the last six months

(excluding regular non-working days): ________ (days)

11. How do you envision your professional future? *(Please tick all appropriate boxes)*

I think that in the near future I will probably…

☐ still work in my profession.	☐ apply for a pension/receive a pension.
☐ not be able to work in my profession.	☐ be unemployed.
☐ search for another job.	☐ be on sick leave.
☐ not be able to work at all.	☐ I don't know yet.

12. Have you been on sick leave due to serious physical illnesses within the last 6 months?

☐ no
☐ yes
